# Secondary hemophagocytic lymphohistiocytosis associated with adjuvant pembrolizumab therapy in a young patient with triple-negative breast cancer: a case report with literature review

**DOI:** 10.1007/s00404-025-08078-4

**Published:** 2025-07-03

**Authors:** G. L. Olmes, M. P. Nigdelis, B. Haj Hamoud, E.-F. Solomayer, M. Bewarder, J. T. Bittenbring, N. Kranzhöfer, L. Thurner, Y.-J. Kim, A. Seibold, M. Doerk

**Affiliations:** 1 Department of Gynecology, Obstetrics and Reproductive Medicine, Saarland University Medical Center, 66421 Homburg, Germany; 2 Department for Oncology and Hematology, University Hospital Medical School, 66421 Homburg, Germany; 3 Institute of General and Special Pathology, University Medical School of Saarland, 66421 Homburg, Saar, Germany

**Keywords:** Hemophagocytic lymphohistiocytosis, Pembrolizumab, Checkpoint inhibitor, Breast cancer, Gynecology

## Abstract

**Purpose:**

Secondary hemophagocytic lymphohistiocytosis (HLH) associated with pembrolizumab is a rare immune-related adverse event (irAE). It features a potential life-threatening status including fever and a hyperinflammatory reaction caused by natural killer cells, CD8 + cytotoxic T cells, and antigen-presenting cells leading to multiorgan failure. Secondary HLH is described for immune checkpoint inhibitor (ICI) therapy. Most descriptions refer to patients with melanoma or lung cancer. We report about a 32 year-old patient with secondary HLH associated with adjuvant pembrolizumab therapy according to the Keynote-522 protocol. It was successfully treated with prednisolone.

**Methods:**

We performed a literature review in PubMed including the terms “HLH OR hemophagocytic lymphohistiocytosis AND breast cancer”. We found four other cases meeting the inclusion criteria (abstract available in English, breast cancer patient, HLH related to ICI therapy).

**Results:**

Apart from the case report, the review featured main aspects about the diagnosis (HScore, histopathological assessment), onset of HLH, and medical treatment. The review indicated that secondary ICI induced HLH in breast cancer patients may be associated with complete response according to their tumor burden. Most cases are reported with an onset of secondary HLH within the neoadjuvant treatment phase and were treated analogously to the Keynote-522 protocol. Our case showed an onset almost 1 year after the initiation of pembrolizumab therapy.

**Conclusion:**

Gyneco-oncologist should be aware of secondary HLH during pembrolizumab therapy and should assess patients with persistent fever using the HScore to diagnose secondary HLH early.

## Introduction

Hemophagocytic lymphohistiocytosis (HLH) is a severe disorder with hyperinflammation by natural killer cells, CD8 + cytotoxic T cells, and antigen-presenting cells, which is still considered to be underdiagnosed [1, 2]. Primary HLH is usually restricted to early childhood and is associated with genetic alterations [2]. Variants in familial HLH (fHLH) genes often affect cytotoxic T and NK cell function by weakening lytic granule formation, secretion, and action [3]. Among the seven fHLH genes, the most common in Europe are *PRF1*, *UNC13D*, and *STXBP2* [2, 3]. Carriers of homozygous or some combinations of heterozygous variants often develop severe HLH in early childhood and require allogenic stem cell transplantation [2, 3]. Secondary HLH usually presents in adults with a mean age of 50 years [1]. It is described in the context of infections, commonly associated with Epstein–Barr virus (EBV) [2–4]. Other forms of secondary HLH are malignancy associated, often with peripheral T cell non-Hodgkin lymphoma, Hodgkin lymphoma, or can occur in patients with a background of a rheumatological disease—most often Still’s disease—what is then termed macrophage activation syndrome [2].

Immune checkpoint inhibitors (ICIs) like pembrolizumab can also trigger secondary HLH [1, 2]. Most data are available for nivolumab, pembrolizumab, and the combination of nivolumab/ipilimumab [5]. Pembrolizumab, which was used in our patient, is standard of care for the treatment of triple-negative breast cancer (TNBC) and especially for early TNBC [6–10]. Pembrolizumab therapy has been associated with various immune-related adverse events (irAEs) such as colitis, hepatitis, and endocrinological disorders, causing severe morbidity requiring specialized multimodal treatment [6–8]. Secondary forms of HLH associated with ICIs are described in the literature even though they are considered rare irAEs [5, 7, 11]. The reported cases of ICI-associated HLH include mostly patients with melanoma, lung cancer, and urothelial cancer [5], while fewer cases have been reported for cases of gynecological malignancies and breast cancer [12–16].

## Case report

A 32-year-old female patient with TNBC under adjuvant intravenous therapy with pembrolizumab 200 mg d1/q21d (11 cycles) complained about fever and arthralgia of the knees and the ankle joints during treatment at the outpatient oncology department (Gynecology, Obstetrics and Reproductive Medicine, Saarland University), Medical Center Homburg, Germany, a tertiary university cancer center.

### Oncologic treatment

The patient (I gravida/I para; cesarean section in 2020) was initially diagnosed with TNBC of the right mammary gland in April 2023 [UICC IIA, cT2 (29 mm), cN0, cM0; estrogen receptor negative (IRS 0/12), progesterone receptor negative (IRS 0/12), HER2/neu negative (Score 0), Ki67 + 30%)]. Genetic testing showed no pathologic alterations of BRCA genes. For fertility preservation, oocytes were cryopreserved and a goserelin acetate 3.6 mg was given subcutaneously d1/q28d before chemotherapy for ovarian suppression. An intravenous chemotherapy according to the Keynote-522 protocol with four cycles of epirubicin 90 mg/m^2^ d1/q14d and cyclophosphamide 600 mg/m^2^ d1/q14d was administered [8]. A febrile neutropenia (Common Terminology Criteria for Adverse Events grade 4) after the first cycle led to a dose reduction for the following three cycles. The chemotherapy was continued with 12 cycles of paclitaxel 80 mg/m^2^ d1/q7d, carboplatin 1.5 area under the curve (AUC) d1/q7d, and 5 cycles of pembrolizumab 200 mg d1/q21d. In January 2024, a breast-conserving surgery with targeted axillary dissection was performed. The histopathologic examination showed a pathologic complete response (ypT0, ypN0 (0/3, sn, i), L0, V0, Pn0). Adjuvant pembrolizumab treatment was continued as recommended by international guidelines [17].

### Clinical presentation

At the initial visit, the patient presented with anemia (hemoglobin 7.7 g/dL; normal value 12.0–15.4 g/dL) and elevated CRP (176.5 mg/L; normal value < 5.0 mg/L). She was admitted to our hospital for 9 days. An intravenous antibiotic therapy with piperacillin/tazobactam 4 g/0.5 g was initiated, and two blood packs were transfused. Chest X-ray, blood, and urine cultures were unremarkable. A gastroscopy excluded upper gastrointestinal bleeding. The rheumatologic screening revealed an elevated rheumatoid factor (104 IU/mL; normal value < 14 IU/mL). A rheumatoid arthritis or ICI-related arthritis was suspected initially; hence, we commenced a therapy with oral prednisolone 25 mg once daily. The treatment led to a normalized CRP (24.1 mg/L) and elevation of hemoglobin (10.2 g/dL). Accordingly, the patient was dismissed without fever and was scheduled for an ambulatory control.

Three days after dismissal, the patient presented back with fever of 39.8 °C and in a reduced physical status. We started an intravenous antibiotic therapy with meropenem 1 g three times daily because of an increased CRP (176.4 mg/L). The echocardiography on the second day was normal excluding endocarditis. An endocrinological assessment revealed no signs for pembrolizumab-associated side effects such as hypo- and hyperthyroidism. The patient remained febrile during this treatment. On the third day, a computer tomography (CT) of the head, abdomen, and thorax showed hepatomegaly with no infectious focus or metastatic disease. The virological examination and the repeated urine and blood cultures were unremarkable. The liver enzymes increased on the third day to GOT 501 U/L (normal values 10–35 U/L) and GPT 178 U/L (normal values 10–35 U/L). The platelets decreased slightly (127000/mm^3^; normal values 140000–400000).

### Diagnostic assessment

The patient was referred to the hemato-oncologic clinic for further evaluation. Given to elevated ferritin levels (89330 ng/mL; normal range 13–150 ng/mL) and a HScore of 284 [18] **(**Table 1**)**, a secondary HLH was suspected. An extensive rheumatologic assessment on the fourth day showed elevated ANA (1:2560; normal range < 1:80), with normal dsDNA antibodies (0.8 U/mL; < 10 U/mL) and elevated SS-A > 240 U/mL (normal range < 7 U/mL) and SS-B 169 U/mL (< 7 U/mL). A bone marrow biopsy was performed at the seventh day. The woman developed pancytopenia with a nadir at the eighth day (leukocyte 2500/mm^3^, normal range 3900–10200/mm^3^), hemoglobin 8.6 g/dL, and thrombocytes 41000/mm^3^.

**Table 1:** HScore adapted to Fardet et al. [18]

HScore items	Selected HScore item	Result of our patient	Mode of assessment	Day
Known underlying immunodepression	no			N/A
Maximal temperature (°C)	Strictly greater than 39.4 °C	39.8 °C		1
Hepatomegaly	Yes		CT	3
Splenomegaly	Yes		Sonography	10
Lower hemoglobin level	Less than or equal 9.2 g/dL	8.6		8
Lower leucocytes count	Less or equal to 5000/mm^3^	2500		8
Lower platelets count	Less or equal to 110000/mm^3^	41000		8
Higher ferritin level (ng/mL)	Strictly greater than 6000 ng/mL	89330		4
Higher triglyceride level (mmol/L)	Strictly greater than 4	4.41		10
Lower fibrinogen level (g/L)	Less or equal to 2.5	0.58		9
Higher SGOT/ASAT level (IU/L)	Greater than or equal to 30 IU/L	501		3
Hemophagocytosis features on bone marrow aspirate	No	Hypoplasia of erythroid stem cells	Bone marrow aspiration and biopsy	7
***HScore***	***284***			
***Probability of having HS (%)***	***99.9***			

HScore of ≥ 250; probability for HLH > 99%

On the ninth day, she developed a hypofibrinogenemia (0.58 g/L; normal range 1.8–4.0 g/d). The performed test for sIL2Ra (2540 U/mL, normal range 158–623 U/mL) was significant. The abdominal sonography on the tenth day confirmed hepatomegaly and a mild splenomegaly. The triglycerides were elevated with 4.41 mmol/L (normal range < 1.69 mmol/L).

Involvement of the central nervous system, eyes, and heart could be excluded after neurologic assessment including a cranial magnetic resonance imaging (cMRI), an ophthalmological examination (fundoscopy) with fluorescence angiography, full coagulation cascade analysis, and cardiologic assessment with electrocardiogram, 24-h electrocardiogram, MRI scan of the heart, coronary artery CT, and echocardiography between the 8th and 23rd day of the second admission. A positron emission tomography on the 11th day showed no residual disease or metastatic burden of the breast cancer.

### Assessment of the bone marrow

The cytological examination of the bone marrow aspirate biopsy showed reduced thrombocytopoiesis, but no signs of hemophagocytosis. The molecular genetic analysis was normal. The histopathological assessment of the bone marrow biopsy revealed a hypocellular bone marrow with hypoplasia of erythroid stem cells and an increased number of macrophages without hemophagocytosis.

### Therapeutic intervention

On the fifth day, a treatment with prednisolone orally 100 mg once daily was started. The ferritin levels decreased rapidly during the prednisolone treatment on the ninth day (8040 ng/mL; normal range 13–150 ng/mL). The body temperature normalized within 2 days after starting prednisolone, and the patient’s status improved. For the treatment of the hypofibrinogenemia, tranexamic acid 1 g 1-0-1 was administered intravenously twice daily from day ten until day 16. For the prevention of corticosteroid-related complications, the patient received oral cotrimoxazole 400/80 mg once daily for prophylaxis of *Pneumocystis jirovecii* pneumonia, pantoprazole 40 mg once daily per os, and vitamin D 20,000 IE once a week. The pancytopenia resolved completely without further transfusions. The patient was dismissed after 24 days of treatment undergoing monthly controls in the outpatient oncology clinic.

### Follow-up

The prednisolone dose was tapered and continued for 6 weeks. Ferritin values normalized (309 ng/mL) and sILR2a decreased to normal ranges (702 IU/mL). After completion of the prednisolone treatment, the patient remained in a good status without signs of HLH relapse. The pembrolizumab therapy was permanently interrupted.

## Discussion

A secondary HLH associated with ICIs is a severe immune-related side effect [19]. The incidence is estimated with 0.4% from a collective of almost 6000 trial patients treated with ICI [11, 15]. The irAEs are common in patients treated with ICI like pembrolizumab [8, 20]. We performed a literature review in PubMed on 25th of February 2025 including the terms “HLH OR hemophagocytic lymphohistiocytosis AND breast cancer”. We revealed 51 results in total. Cases were only included if they featured an abstract available in English, the patient had breast cancer, and the HLH was related to ICI therapy. Four cases met the inclusion criteria **(**Table 2**)** [13–16]. We compared the extracted data based on the year of publication, patient’s age, intrinsic subtype, UICC stadium, ICI, major symptoms, HScore, histopathologic confirmation, onset of HLH after the induction of ICI therapy, tumor response after therapy, other irAEs, therapy, time to recovery, and follow-up **(**Table 2**)**. The literature review is restricted only to breast cancer patients treated with pembrolizumab and reveals, therefore, limitations according to patient collectives, ICIs, and treatment regimes.

**Table 2:** Literature review in PubMed including the terms “HLH OR hemophagocytic lymphohistiocytosis AND breast cancer”

Author	Year of publication	Patient’s age (years)	Intrinsic subtype	UICC stadium	ICI	Major symptoms	HScore	Histo-pathologic confirmation	Onset of HLH after induction of ICI therapy (days)	Tumor response after therapy	Other irAEs	Therapy	Time to recovery (days)	Follow-up (months)
This case	2025	32	TNBC	IIA	Pembrolizumab	Fever	284	No	356	Pathological complete response	Arthritis, abnormal rheumatologic screening	Prednisolone	19	5
Walmsley et al. [15]	2025	35	TNBC	IIIC	Pembrolizumab	Fever	193	Yes, bone marrow biopsy	166	Residual tumor burden	N/A	Dexamethasone	9	12
Kawamura et al. [14]	2025	38	TNBC	IIIC	Pembrolizumab	Fever	199	Yes, liver biopsy	114	Pathological complete response	Colitis	Pulse steroid therapy MMF, cyclosporine, tocilizumab	19	N/A
Patton et al. [16]	2024	40	TNBC	IIIA	Pembrolizumab	Fever	98%−99%	No	92	Radiographic complete response	No	Methylprednisone, etoposide, tocilizumab	23	N/A
Al-Samkari et al. [13]	2018	58	Switch to TNBC in metastatic disease	IV	Pembrolizumab	Fever, rash	254	N/A	84	Interval regression	Hypophysitis	Methylprednisolone, etoposide	30	24

The first breast cancer patient with secondary HLH is reported in 2018 [13]. The other cases are dated from 2024 to 2025 [14–16]. The mean age of the patients identified by the review was 42.75 years (range 35–58 years) [13–16]. The patient in our case was aged 32 years and was younger than the average. All reported patients with ICI-associated HLH were female and had TNBC [13–16]. For the case reported by Al-Samkari et al., a receptor switch to TNBC is described for the metastatic disease [13]. Comparing our review with a data set by Diaz et al., which included 190 cases of ICI-associated HLH, they included mainly melanoma or lung cancer patients (*n* = 118, 62.1%) [5]. That collective revealed a median age of 64 years with a tendency for male sex observed (*n* = 118, 64.8%) [5] and was, therefore, different from the reported cases of our review. Patients with TNBC are often of younger age compared to other intrinsic subtypes and receive a chemotherapy more often in early breast cancer [21–24].

The UICC stadium in our patient was IIA; for three others, it was reported with UICC IIIA and two with UICC IIIC [14–16]. Only the case by Al-Samkari et al. revealed a metastatic setting (UICC IV) [13]. The patient in our case was treated with pembrolizumab according to the Keynote-522 protocol [8] as well as the three other cases extracted from the review [14–16]. The patient with UICC IV received pembrolizumab in combination with eribulin in the context of a trial (NCT02513472) [13].

On their admission to our hospital, the patient complained about fever. Fever as a major symptom on the initial visit is reported for all cases in the literature review [13–16]. In addition, rash was reported by Al-Samakri for metastatic patient at the initial visit [13].

Clinical criteria for HLH are summarized in the HLH-2004 criteria that comprise eight items, i.e., fever, splenomegaly, bicytopenia, hypertriglyceridemia and/or hypofibrinogenemia, and hemophagocytosis, low/absent NK-cell-activity, hyperferritinemia, and high-soluble interleukin-2-receptor levels [25]. Five of them have to be fulfilled for the diagnosis of HLH [25]. Our patient showed seven out of eight items excluding NK-cell-activity, which she was not tested for. Fardet et al. validated the HScore to estimate the risk of reactive HLH including underlying immunosuppression, high temperature, organomegaly, triglyceride, ferritin, serum glutamic oxaloacetic transaminase, and fibrinogen levels, cytopenia, and hemophagocytosis features on bone marrow aspirate [18]. Our patient’s score was 284 marking a probability of 99.9% for HLH (Table 1). According to Fardet et al., a cutoff value for the HScore of 169 received a sensitivity of 93% and a specificity of 83% for the correct diagnosis of HLH [18]. The HScore was mentioned for all reported cases and indicated a high probability for HLH, suggesting a fulfillment of many clinical criteria for HLH [13–16].

A histopathological confirmation of hemophagocytosis is reported for two cases and was detected by a bone marrow biopsy and a liver biopsy [14, 15]. The present case did not show signs of hemophagocytosis in the bone marrow biopsy. HLH can be diagnosed by histopathological evidence of hemophagocytosis in the bone marrow biopsy, which is pathognomonic, but not required for the diagnosis [1, 26]. In 85% of HLH in adults, hemophagocytosis is histopathologically confirmed [1]. In cases with ICI-associated HLH, hemophagocytosis is described for 46.5% [5]. The review might reflect this aspect with two reported cases including histopathological proof of HLH.

The average time from the onset of ICI therapy to first symptoms of secondary HLH in the reported collective was 114 days (range 84 days to 166 days). In comparison, our case had a longer duration than the average. Comparing our case to the data from Diaz et al., the mean time to onset of symptoms has been described as 102 days and median 50 days [5]. Therefore, our case might show an unusual late onset of HLH compared to the literature review and to Diaz and colleagues. The onset of symptoms of HLH in the cases treated analogously to Keynote-522 protocol [8] began for the reported cases during the neoadjuvant treatment period [14–16], whereas our case was under adjuvant treatment of pembrolizumab.

A retrospective analysis of the dataset of the Keynote-522 trial stated out that irAEs correlated with pathological complete response [27]. Our patient also showed a pathological complete response after neoadjuvant treatment with pembrolizumab. For two of the three cases treated according to the Keynote-522 protocol, the review showed a pathological or a radiographic complete response [8, 14, 16]. The other one showed residual tumor burden [15]. The metastatic patient revealed also an interval response through ICI therapy [13].

The review mentioned two other cases with irAEs: colitis and hypophysitis [13, 14]. The presented case demonstrated also irAEs including arthritis and an abnormal rheumatologic screening. Autoimmune diseases can also trigger secondary HLH [1, 25]. Patients with preexisting autoimmune-like rheumatoid arthritis disease can develop a flare-up in 28% during ICI therapy, as an analysis of medical records demonstrated by Alexander et al. [28]. Our patient showed signs of arthritis and revealed elevated rheumatoid factor, ANA titer, and SS-A and SS-B. Arthritis is described as an irAE of pembrolizumab therapy and also in the context of HLH [20, 29]. According to elevated ANA in our patient, a prospective study in 152 patients with solid tumors treated with ICI demonstrated that most patients with severe irAEs had changes in the ANA titer before the onset of the irAE [30]. We suggest for our patient that ANA titer was either elevated because of the secondary HLH as an irAE or in the context of elevated SS-A and SS-B, implying a preexisting autoimmune disorder.

As differential diagnosis for secondary HLH, other irAEs are considered for discussion [13, 14]. Al-Samkari et al. report a patient with metastatic breast cancer treated with pembrolizumab, who developed HLH and showed signs of hypopituitarism related to pembrolizumab [13]. We performed a comprehensive diagnostic assessment including cardiological, neurological, ophthalmological, and endocrinological diagnostics. Our patient did not show other signs for irAEs apart from arthritis.

An update of the EULAR/ACR recommendations on treatment for HLH was published 2023 [3]. General aspects of the therapy of HLH include immunosuppressive treatment, discontinuation of trigger factors, and supportive therapy [3]. A context-oriented therapy is necessary, and an expert in hemato-oncology should be consulted, if HLH is suspected [3]. We treated our patient with corticosteroids for 8 weeks revealing a complete response of HLH, and stopped pembrolizumab therapy permanently. The supportive therapy should contain bacterial, fungal, and viral (HSV/CMV) prophylaxis, proton pump inhibitors, and vitamin D [3]. The treatment containing corticosteroids is recommended for all forms of HLH and is considered for moderate forms of secondary HLH [2, 3]. The review indicated another case treated only with corticosteroid, i.e., dexamethasone [15].

For primary or severe secondary or progressive forms of HLH, more intensive immune suppressive/modulatory treatment approaches are often necessary [3]. The HLH-94 protocol is used for severe forms of secondary HLH and contains corticosteroid and etoposide as main components [31]. One case was treated with corticosteroid and etoposide [13]. In patients with severe HLH, medical treatment with JAK-inhibitors, IV immunoglobulin, tocilizumab (αIL-6R) or anakinra (αIL-1R) is also described additionally to etoposide-based regimes [31]. For the case reported by Patton et al., therapy with tocilizumab was necessary after HLH progression on corticosteroid and etoposide treatment [16]. Another treatment regime is described for the case reported by Kawamura et al. including pulse corticosteroid therapy, mycophenolate mofetil (MMF), cyclosporine, and tocilizumab [14].

The mean time until recovery referred to the review was 20 days (range 9–30) [13–16]. Our patient needed 19 days until recovery which is in line with the reviewed cases and with Diaz et al., who estimated recovery within 20 days [5]. Our patient was followed up until 5 months after discontinuation of the corticosteroid therapy. For two cases, a follow-up of 12 and 24 months was mentioned [13, 15].

## Conclusion

With the widespread use of pembrolizumab in TNBC, the relevance of severe irAEs like secondary HLH is increasing. For the diagnostic workup of persistent fever in these patients, the HScore should be assessed early by the gyneco-oncologist. Our case showed a long delay from treatment begin with pembrolizumab to the onset of HLH, implicating the necessity to perceive ICI-associated HLH also in the adjuvant treatment phase. The literature review suggests that patients with TNBC who develop a secondary HLH might reveal a pathological complete response.

## Data Availability

No datasets were generated or analysed during the current study.
